# Pathogen Reduction of Transfused Blood Components—The End of the Beginning Rather than the Beginning of the End

**DOI:** 10.3390/pathogens15040442

**Published:** 2026-04-20

**Authors:** Albert Farrugia, Laurence Corash, Raymond Goodrich, Leni von Bonsdorff

**Affiliations:** 1 School of Biomedical Sciences, The University of Western Australia, 35 Stirling Highway, Crawley, Perth, WA 6009, Australia; 2 International Plasma and Fractionation Association, 1066 CX Amsterdam, The Netherlands; leni.bonsdorff@ipfa.nl; 3 Department of Laboratory Medicine, University of California, San Francisco, CA 94143, USA; lcorash@cerus.com; 4 Department of Microbiology, Immunology, and Pathology, Colorado State University, Fort Collins, CO 80523, USA; ray.goodrich@colostate.edu

**Keywords:** blood safety: emerging pathogens, pathogen reduction

## Abstract

Therapeutics derived from donated blood or its constituents are classifiable into blood components and plasma derivatives. The latter are defined as medicines/drugs/pharmaceuticals produced from the industrial fractionation of thousands of pooled plasma donations and characterised with relative precision to a pre-defined specification through sampling of a homogenous pharmaceutical batch. The former are defined as components/biologicals produced using relatively simple (but increasingly complex) technologies in blood centres from single or small pools of isolated components from whole blood and are pre-specified through regulatory standards with relatively wide limits because of the inherent biologic variability of individual donors. This review discusses the evolution of technology to reduce the risk of pathogen transmission by blood-derived therapeutics, assess the state of the approved technologies for pathogen-reduced blood components, and examine the features of the blood-provider and regulatory framework globally that have shaped, and in some instances impeded, the implementation of component pathogen reduction to an extent equivalent to that achieved for plasma derivatives. The ensuing risks to the public’s confidence in the blood supply are discussed, and remedial actions are proposed. The features of a new paradigm for blood safety are outlined.

## 1. Introduction

**Figure 1:**
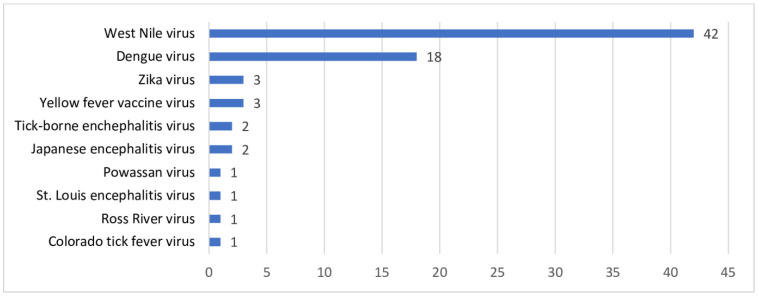
Globally reported transfusion-transmitted arbovirus cases. From [2].

Although rare, the transmission of infectious agents, particularly but not exclusively emerging pathogens, including arboviruses, still occurs in the recipients of blood bank components, including red cells, platelets and plasma [1,2,3] (Figure 1) [4]. These pathogens pose a risk that, in certain geographies, is possibly underestimated [5]. Established pathogens, including Hepatitis A and parvovirus B19 [B19] [6]; Hepatitis E [7]; malaria (Table 1) [8,9,10,11]; *T. cruzi* [12]; and, in the low- and middle-income countries (LMICs), the historically important Human Immunodeficiency Virus (HIV), Hepatitis B Virus (HBV) and Hepatitis C Virus (HCV) [13,14,15], are still transmitted.

The rare, but detectable, occurrence of transfusion transmission infections in high-income countries (HICs) should also be of concern (Table 2 and Table 3) [16,17]. In particular, the continued incidence of Transfusion Transmitted Bacterial Infection (TTBI) is a problem with a long-recognized history, for which measures have been developed without being universally implemented. Early case reports in 1971 showed that as many as 2.5% of individual platelet concentrates stored at room temperature were contaminated with bacteria, and transfusion to some patients resulted in immediate septic reactions [18]. Despite additional measures, such as an initial bacterial culture to reduce the risk of TTBI, the incidence of delayed septic transfusion reactions reported by passive surveillance persisted and remained underestimated [19]. Despite the publication of nine prospective studies demonstrating the problem over the next thirty years [20], the sector did not enact mandatory safety measures until the American Association of Blood Banks (AABB) changed the accreditation standard to require bacteria risk mitigation [20]. Subsequently, another ten years elapsed before the US Food and Drug Administration published draft guidelines addressing the issue in 2014, which was implemented in 2019. The risk of bacteria in whole blood, plasma and red cells has never been extensively examined, but cultures of these blood components from healthy blood donors have demonstrated bacterial contamination, especially in donors with periodontal disease [21,22].

In contrast, transmission of pathogens by industrially manufactured plasma-derived medicinal products (PDMPs), including coagulation factors and immunoglobulin, has not been recorded in the established markets for the past thirty-five years. An analysis of the chronology of the introduction of blood safety measures over the 1980s–90s shows that while the safety of the mainstream transfusion of blood and fresh components was improved significantly by the introduction of donor selection procedures and screening tests, the pivotal measure that rapidly reduced viral transmission through PDMPs was, and remains, the inclusion of virucidal steps in the manufacturing process [23,24,25]. Rigorous processes, such as severe heating, solvent–detergent treatment and nano-filtration, ensured that any agents that entered the large pools of donations needed for the industrial fractionation of plasma were inactivated and/or removed. This led to a reversal of the earlier situation whereby the risk of infection was much higher for recipients of PDMPs relative to that for transfusion recipients.

## 2. The Introduction of Pathogen Reduction (PR) for Blood and Fresh Components

In the early years of the HIV epidemic in the USA, particularly in San Francisco, over 1% of blood issued for transfusion was contaminated [26], which focused public attention on the safety of blood transfusion. While the risk of hepatitis was well-recognised and was steadily reduced through successive generations of selection and screening procedures [23], the AIDS disaster provided an impetus towards the adoption of new technologies, particularly Nucleic Acid Testing (NAT), and contributed to an aspiration for “Zero Risk” in blood transfusion. Surveying the transfusion landscape in the early 1990s, a significant residual risk for the transmission of the historically important TTVs was still present [27]. The greatest contributor to this risk, the serologically silent window period, has been mostly obviated by the inclusion of NAT in the blood-screening paradigm so that the corresponding data in the current era indicates a much lower residual risk of transmission [28]. Table 4 summarizes these contrasting situations. The implementation of successive generations of NAT—mini-pool (MP) and single donor (SD)—is the main source of these enhancements [29]. Importantly, it required 34 years of continued scientific evolution of NAT to achieve these low risk levels in 2025.

Early efforts to introduce pathogen reduction (PR) measures, analogous to the highly successful measures of the PDMP industry, focused on diminishing this residual risk through inactivating the infectious agents [30]. A number of parallel endeavours, mostly involving photochemical treatments of cellular components, were developed over the succeeding years, and have been introduced in a number of HICs, as well as in one LMIC (Honduras) [31]. The reader is referred to a number of excellent reviews on the established technologies that have been approved by the regulatory authorities [31,32,33,34]. Table 5 summarizes the main technologies [35]. Plasma and plasma-derived components, such as cryoprecipitate, have also been introduced in processes using these photochemical techniques [36,37]. The amenability of these plasma components to solvent–detergent (S/D) treatment has resulted in this technique’s wide adoption, resulting in batch-manufactured, pharmaceutical therapies [38,39]. As of this writing, licensed forms of PR plasma, cryoprecipitate and platelets are widely available, and the continuing clinical validation for PR red cells [40] is anticipated to result in the approval for these products in the near future. Efforts to PR whole blood (WBPR), which is perceived as being particularly beneficial for LMICs [41], have resulted in a widely cited clinical trial (the AIMS study) suggesting utility in decreasing transfusion-transmitted malaria [42]. Subjects in this trial were monitored for coagulation and platelet parameters, with no abnormalities being found in the group transfused with PR WB, but demonstration of substantial damage to platelets and plasma proteins in PR WB with another technology suggests that more studies are needed before this highly desirable product can become a reality [43,44]. The results of the ongoing Mirasol Evaluation of Reduction in Infections Trial are awaited [45].

## 3. The Emergence of More Infectious Risks

**Figure 2:**
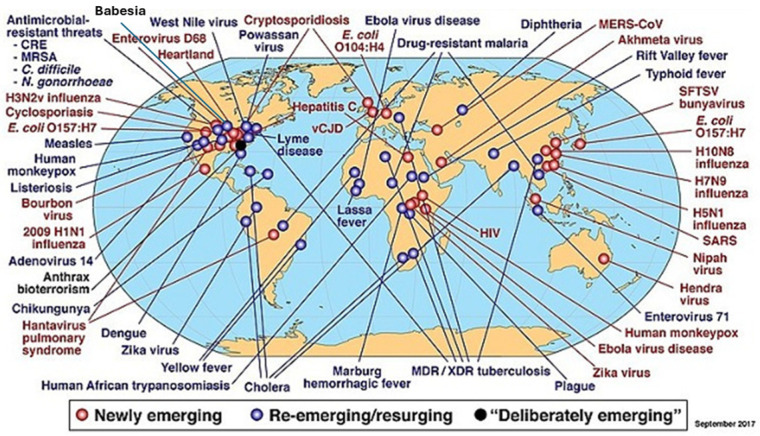
Global examples of emerging and reemerging infectious diseases. Reproduced from [55].

The early years of PR technology (PRT) focused on the historically recognized transfusion-transmitted viruses (TTVs). With the new millennium, a succession of other transfusion-transmitted infections gained visibility. These included long-recognised parasitic risks, such as transfusion-transmitted malaria [46], and other parasites, such as Chagas disease [47], babesiosis [48] and other vector-borne infections. In addition, emerging TTVs have included West Nile Virus [49], dengue [50], hepatitis E [51] and others [4]. Man-made environmental and behavioural factors are mostly responsible for the shifts in vectorial geographical distribution behind these infectious diseases’ emergence and re-emergence [52,53]. The possible evolution to more infectious forms that will then infect vectors will enhance these risks [54]. Figure 2 includes a non-exhaustive list of these infections [55]. Many of these infections have been shown to be not transmissible through blood and component transfusion, e.g., HINI Influenza, Severe Acute Respiratory Syndrome (SARS) and COVID (SARS-CoV-2), although the World Health Organisation adopts a cautious position regarding the possibility of transmission of some of these infections [56].

While the widespread transmission of West Nile Virus (WNV) led to mass screening regimens with NAT, ranging from universal screening in the USA [57] to more selective strategies in other geographies [58,59]; most of these infectious threats have been addressed through increasingly complex criteria that are specified in donor questionnaires and seeking to defer donors who have visited the areas where the infections have been prevalent. These deferral measures have a significant and deleterious effect on the propensity of donors to return [60,61]. In situations where widespread deferral and/or screening was impractical, selective use of PRT was used to provide blood components, notably platelets [62,63].

Although overshadowed by concerns over other pathogens, sepsis following the transfusion of bacterially contaminated blood was an early concern [64,65]. These concerns were allayed by the rarity of such events following the introduction of closed disposable plastic bag systems for blood collection and processing. Following the development of platelet concentrates stored at room temperature, which is required for the preservation of platelet post-infusion kinetics, studies indicated that up to 10% of platelet concentrates were contaminated with bacteria [66], while a survey from the USA reported that 10% of transfusion-associated fatalities were due to bacterial contamination [67]. These concerns led to the introduction of a number of mitigating factors to minimize the risk, including the diversion of the first few milliliters of a blood donation to exclude the most heavily contaminated portion; the standardization of donor arm disinfection techniques; and, in a number of countries, the testing of platelets by bacterial culture before release to exclude bacteria-containing units [68]. The near-misses reported by one haemovigilance system, involving bacteraemic platelet transfusions from culture-negative units [69], suggests that this battery of measures does not fully eliminate the bacterial contamination risk. A number of countries in Europe [70], North America and elsewhere have implemented PRT for platelets. Risk mitigation with PRT is not complete, as reports have indicated that viral transmission [71] and bacterial transmission [72] may occur with PRPC.

## 4. Pathogen Reduction—Why

By the beginning of the new millennium, the viral transmission risk for recognised pathogens transfused with blood components in the USA had diminished through the use of increasingly sensitive screening tests to a level that could be better quantified through mathematical modelling, as the transmissions detected from epidemiological surveillance were vanishingly small, although, rarely, such events were recorded [73]. The blood system responded rapidly to the threat of the newly emerged WNV epidemic, although not before transmissions occurred. Because of the rarity of these events, the impetus to introduce PR for blood components, relative to the establishment of processes for PDMPs, was somewhat muted. Parallel concerns that continue to drive the development of PRT are discussed in the following subsections.

### 4.1. Risk of Transmission of the Established Pathogens

The blood-screening regimens used in HIC are virtually unavailable in many LMICs, and the residual risk of the transmission of HIV, HBV and HCV remains high [13,74,75,76,77]. The introduction of more sensitive screening tests, such as NAT platforms capable of delivering a lower residual risk than many of the technologies used in these countries [78], may decrease transfusion risks, as may the development of donor selection questionnaires, with varying results [79]. Such testing + selection approaches have also been proposed for minimising the risk of transfusion-transmitted malaria [80]. A Canadian study that modelled the residual risk for transmitting HIV, HBV and HCV through blood components concluded that PRT significantly reduces the residual risks of transfusion transmission of these viruses and could enable the removal of blood donor deferral criteria for sexually risky behaviours [81]. A review on the residual risks of bacterial contamination for pathogen-reduced platelet components shows that the timing of pathogen inactivation strongly influences the efficacy of PRT, as does whether the pathogens are PR-resistant spore-forming species [82].

The use of a robust PRT may lend itself to a form of technological leapfrogging, such as has occurred in the development of telephonic communication [83,84,85], removing the need to implement, partially or fully, screening systems in lieu of a universal PRT that can be used for all blood components and whole blood. Although such a PRT is not yet available, leapfrogging the need to screen for bacteria and emerging arboviruses and to irradiate platelets has already been implemented in some countries [31]. The introduction of available technology should also decrease the incidence of transmissions such as those recorded recently in India [86]. The cost of PRTs, which is augmented by the need for GMPs around the use of PRTs, remains problematic for LMICs, but cost-effectiveness could be improved if measures to counter additional risks, such as Graft Versus Host Disease (GVHD), bacterial sepsis and emerging pathogens, which are currently unmitigated in these countries, are included in the measures that could be leapfrogged by PRT [87], as well as discontinuing certain donor deferral measures [81].

### 4.2. Risk of Emerging Pathogens

Reiterating the experience of the past half-century, pathogens that have emerged to cause transmission through transfusion of blood components have not infected the recipients of plasma derivatives. This includes WNV infection, which was transmitted to many recipients of fresh components before molecular diagnostics established the current screening system. Patients have been infected by WNV-negative blood components following the implementation of screening by MP NAT [88] and ID NAT [89], indicating that NAT, while significantly enhancing blood safety, has limitations. It should be noted that the United States Food and Drug Administration (FDA) and the United States Pharmacopeia (USP) do not mandate WNV screening of source plasma for manufacture [90], following demonstration by the plasma industry that the viral inactivation steps introduced in the process inactivate the virus [91] despite evidence of WNV contamination in plasma pools [92]. Currently available PRTs inactivate a wide range of newly emerged TTVs, including WNV, Dengue virus, Chikungunya and Zika [2]. In LMICs, which have been particularly stricken by these agents [62,93,94], as well as in HICs, the use of PRT enabled the maintenance of the blood component supply chain during epidemics.

### 4.3. Streamlining Donor Selection and Screening

The risk of emerging pathogens has been partially mitigated in most systems by donor selection aimed at temporarily deferring donors who have travelled to endemic areas [95] and through the selective screening of donations using an available methodology [4,96]. Studies have assessed the possibility of substituting these increasingly complex and expensive measures with a PR process that can substantially reduce the risk of transmission of established, emerging and re-emerging pathogens [81,97]. These studies suggest that testing for emerging pathogens, as well as the deferral of donors currently deferred for risk behaviours, can be removed if PRT can be implemented for all transfused components. This possibility is supported by regulatory guidance exempting platelets that have undergone PR from the need to be tested for bacterial contamination [98]. The elimination of some selection processes should enhance the supply of blood components from donors who would otherwise not return as the result of deferral [60,61,99]. In this context, it is reasonable to propose continued testing for viruses that are resistant to current PRTs, such as Hepatitis E Virus (HEV) and Parvovirus B19 (B19), while discontinuing part or all of the testing paradigm for susceptible agents, including bacterial testing and cytomegalovirus (CMV) serology, as is currently approved by regulators. Stramer et al. have presented data on pathogen loads of the West Nile (WNV), dengue (DENV), Zika (ZIKV) and Chikungunya (CHIKV) viruses; Babesia microti; Trypanosoma cruzi; and Plasmodium to support replacing testing for these vector-borne pathogens with effective pathogen-reduction technologies [97]. Busch et al. have proposed modified testing strategies in combination with effective pathogen-reduction technology for HIV, HBV and HCV by limiting testing to MP NAT rather than ID NAT while discontinuing serological screening to improve the cost efficiency of preventing transfusion-transmitted infections [100]. Further streamlining of the platelet supply chain can be effected by storing PRPC for seven days when using some forms of PRT [72,101,102,103], as is specified in some quality standards [104].

### 4.4. Decreasing Non-Infectious Transfusion Risks

A wide-ranging haemovigilance review of the experience of the French transfusion system since the introduction of universal PRT of platelets in 2018 [105] indicates that PR platelets are associated with significantly lower adverse transfusion reaction rates compared with earlier generations of platelet products. Significantly, expiry rates also decreased.

Transfusion-associated GVHD is a rare but severe complication of blood transfusion that is historically addressed through gamma (γ) irradiation of components to inhibit residual donor T-lymphocyte proliferation [106]. PR of platelets leads to equivalent or superior results compared with γ irradiation [107] and similar results have been reported for PR red cells compared with irradiated, washed and stored red cells [108]. Current processes for transitioning caesium γ irradiators to X-ray instruments may need to consider the possibility that for blood banks, this might lead to a technology that may be redundant by the time that PRT is embedded in transfusion practice [109], given that the FDA has also exempted PR platelets from the need to be γ irradiated to mitigate GVHD risk [98].

## 5. Pathogen Reduction—Why Not

The delay in implementing PR has been attributed to the factors discussed in the following subsections.

### 5.1. Costs and Cost-Effectiveness

With the increasing scope of the blood safety paradigm and its consequences in the form of expensive measures such as NAT, a body of opinion mostly embedded in the public health and academic sectors has utilised the traditional techniques of pharmacoeconomics to analyse the cost effectiveness (CE) of these measures [110,111,112,113,114]. Most of these analyses (CEAs) concluded that the measures, all of which are now embedded in transfusion practice, were not cost-effective according to the criteria and practice of funding bodies, and therefore, should not be implemented. Similar work has assessed the CE of PRT in various scenarios [87,115,116,117,118], most of which reach the same conclusions as had been reached for other blood safety measures, although a number of qualifying factors influence the final outcome. The substitution of safety measures such as gamma/x-ray irradiation for Transfusion Associated GVHD, bacterial screening, CMV serology, malaria deferrals, extension of PC storage in some countries, and use of MP NAT as opposed to ID NAT for WNV need to be factored into the CEA for PRT of PC. The critical issue is to calculate the net cost impact of PRT to replace existing safety interventions. In addition, in jurisdictions such as the USA, which currently use largely PR apheresis platelets, transitioning to PC manufactured by the whole blood buffy coat method with PRT can lead to considerable cost savings and elasticity of the platelet supply, as has been experienced in Canada [119,120]. Measures to assure the safety of previously frozen quarantine plasma for transfusion, by the use of PRT to safely issue male, and especially AB male, quarantine plasma [121] which has not been qualified by a second round of viral screening, contribute to its cost-effectiveness. The inclusion of PRT for RBCs, which was unapproved at the time of most of these studies, was shown to significantly improve the CE of available PR platelets and plasma [81,98]. Red cell PRT is approved in Europe through Conformité Européenne marking [122] and has been clinically validated [40]. The emergence of a new pathogen influences the CE of PR platelets and PR plasma [87], the extent of which depends on the nature of the pathogen. A similar analysis for PR platelets resulted in a high Incremental Cost-Effectiveness Ratio (ICER), depending on the time interval between the initiation of PRT and the emergence of a new virus [123]. This study criticised an unpublished cost-effectiveness analysis (CEA) developed by a PRT manufacturer to support advocacy for the technology’s adoption and reimbursement for assuming that an emerging virus would already be present in the blood supply. It is uncertain as to how this scenario, which mirrors the situation encountered with many blood-borne viruses, is unreflective of the realities around blood safety.

Irrespective of the merits of the various CEA, many of them disregard the public’s unwavering aversion to any form of risk from blood transfusion [124]. This is well-articulated in the report of the recent IBI from the UK, which heavily criticised its perceived delay in the implementation of blood safety measures, such as blood screening for HIV and HCV, and viral inactivation of plasma products [125]. The perception of the patient, rather than that of the health economist, should influence blood safety policy [126]. The public’s willingness to pay for enhanced healthcare is high when directed to prevention, which would include blood safety measures, rather than treatment of harms [127] and is higher in higher income and highly educated social groups [128], a demographic that is more likely to include blood donors [129].

### 5.2. Limited Capacity to Inactivate Pathogens

Current PRTs have limited capacity to inactivate some non-enveloped viruses, bacterial spores and infectious prions [71,130,131,132,133], although this varies between technologies [133]. A significant capacity to inactivate bacteria [115] does not preclude the possibility of infections [72], albeit at a considerably reduced rate [131]. There is no issue around prions, as no biological inactivation process is effective against these agents, and the risk of transfusion transmission is negligible [134].

Hence, it is reasonable to propose that the current technologies are less robust than those used in the plasma industry, whose efficacy is demonstrated through several decades of zero transmissions by products derived from large pools of plasma and administered on a chronic basis, which would have infected recipients in the absence of viral inactivation [135]. These are limitations demanding attention if the promise of PRT is to be realised. They should not be used to delay the implementation of PRT whenever this is possible—the perfect should not impede the good, as has been pointed out by diverse authorities on blood safety:

“Where uncertainties or countervailing public health concerns preclude completely eliminating potential risks, the FDA should encourage, and where necessary require, the blood industry to implement partial solutions that have little risk of causing harm” [136].

### 5.3. Quality of PR Blood Components

Studies indicate that PR plasma [137], PR red cells [138] and PR platelets [139] have a number of abnormalities in their in vitro properties compared with untreated components. These have affected some aspects of the safety and efficacy of these products. In the United States, a series of significant thrombotic incidents associated with transfusions of a specific PR (solvent–detergent-treated) plasma resulted in its withdrawal from the market [140]. Variation of the manufacturing method obviated this problem, and the product continues to be used with a high level of safety. Use of PR red cells led to the formation of red cell autoantibodies, mediated through the immunogenicity of the PR chemicals [141], leading to a revision of the PR process in an effort to minimise this effect, with only partial success [40]. These effects on in vitro and in vivo red cell properties have not been reflected in significant clinical effects, such as haemolysis from the autoantibodies and shortened red cell survival [142,143,144]. Investigation of antibodies in recipients of PRT RBC have shown that survival was not substantially impacted, suggesting that not all antibodies are physiologically active. These observations require extended post-marketing studies for definitive evaluation of both native and acquired antibodies that may impact PRT RBC transfusions. Studies on PR platelets summarised in a systematic review [145] noted a lower platelet count increment (CI) and a lower corrected count increment (CCI), as well as higher rates of platelet refractoriness, alloimmunisation and a higher number of platelet transfusions in patients given PR platelets. It is noted that the decreased CCI following PR of platelet concentrates are not accompanied by any evidence of lung injury in patients, based on large clinical multicentre studies comparing them with conventional platelets [146]. The incidence of acute pulmonary injury and mechanical ventilation was significantly reduced in patients with hematologic and oncologic disorders requiring extended platelet support. These observations indicate a potential benefit of PRT to patients and healthcare systems beyond prevention of transfusion-transmitted bacterial infection [109].

The PDMP industry has had to resolve similar issues in the evolution of virally inactivated PDMPs. Early efforts to heat Factor VIII (FVIII) concentrates resulted in some instances of neo-antigenicity to FVIII, decreasing the products’ efficacy [147], while heating the prothrombin complex led to the generation of thrombin and thrombogenicity in animal models [148]. Other viral inactivation steps, such as the pasteurisation of albumin [149] and the acidification of immunoglobulin [150], also affect the proteins involved. The plasma industry has continued to refine these viral inactivation methods to ensure the viability of the products. A similar process of continuous improvement is required by the manufacturers of PRT. In the interim, the alterations in product safety and efficacy, although important, have not resulted in clinical effects that should impede the implementation of the current generations of PRTs.

## 6. Summary Reflections

Based on the issues specified in the present work, the patchy introduction of PRT globally should concern physicians practicing clinical transfusion medicine and the wider public health environment. Importantly, when asked, patients assume that blood products for transfusions will be safe and available when needed. This has not always been the case. These issues, and similar perceptions, shaped the thinking of the plasma industry and its attendant overseers in the period preceding the introduction of viral inactivation. The delayed introduction of viral inactivation because the first efforts did not eradicate all viruses, the subsequently partially justified apprehensions that viral inactivation would lead to neo-antigenic therapies, and supply issues, which in the final analysis, came down to costs and prices, were all matters that the Infected Blood Inquiry (IBI) considered. None of these arguments were considered valid [125], and the IBIs condemnation of the participants of the system at that time received quasi-universal endorsement in the legal, political and patient sectors, and, above all, in the court of public opinion.

This burden of experience, increasingly viewed with detachment, causes one to reflect on the corporate memory of the public health sector in particular in regard to blood safety. Several studies have attempted to generate mathematical models to fit empirical data on a range of events into an analytical tool that was designed to delineate the length of the collective memory for cultural events in general [151,152,153]. This work suggests that the average attention received by cultural products, which we suggest includes the vast body of work addressing blood safety problems in the 1980s–90s, decays following a universal biexponential function, with the longest retention in communicative memory being 20–30 years [153]. Given the relative diminishing in public and media interest in blood safety from ca 2000, the fading of the lessons learned in the 1970s–1990s is understandable. This is augmented by the increasingly frail financial commitments to blood safety by established agencies [154]. The constraints placed on the WHO’s budget through the withdrawal of the USA and some other countries has led, amongst other cuts, to the removal of the Blood Safety Division and the abolition of the co-ordinator’s position (Maryuningsih Y personal communication 2025).

**Figure 3:**
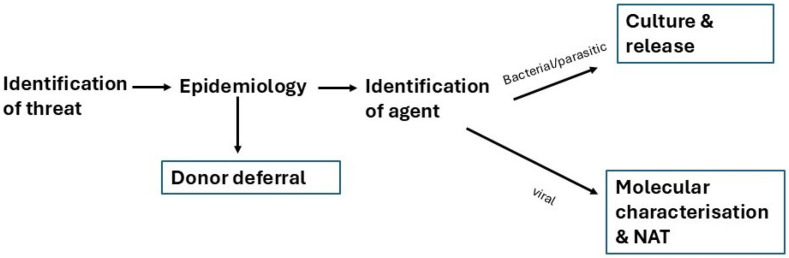
Linear representation of the conventional approach to blood safety threats.

The resultant complacency relies, to some extent, on a linear perception of the management of blood safety threats illustrated in Figure 3. This linear approach is demonstrably deficient [155]. Apparently unrelated changes in the ecosystems around pathogens, often related to environmental destruction, international commerce and travel, and others [52], have resulted in unpredicted and unpredictable threats to blood safety. In some instances, these threats have ensued from modifications in blood bank practice, including measures to counter safety threats [155,156]. The linear progression referred to above has been based on the empirical management of the threats encountered in the past decades, which has included a rapid awareness of the epidemiology of emerging threats, which is itself dependant on a short incubation period preceding overt illness, and an equally rapid molecular characterisation of the infectious agent. In the hypothetical situation of an absence of these characteristics, the linear model collapses and the blood supply will be infected with a potentially transmissible agent before any measures could be implemented.

**Figure 4:**
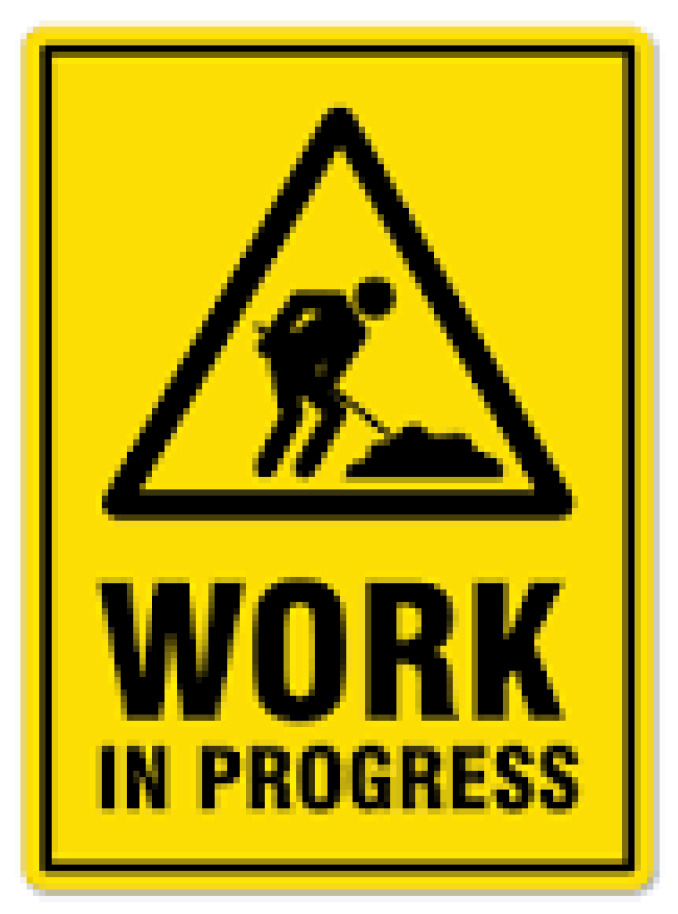
Pathogen reduction technology—a work in progress.

This is not to say that implementation of pathogen reduction would eliminate this risk fully. Again, hypothesising an agent resistant to current PRTs, such as a new non-enveloped virus with epidemiological, clinical and structural features that render it resistant to conventional selection and screening measures—e.g., a long incubation period and asymptomatic for infection—the situation for clinical transfusion would be grim. Furthermore, as exemplified in this work, PRT does not fully mitigate the risk of transmission of current TTIs. Hence PRT is a work in progress, and the companies involved need to maintain their efforts, with the gold standard established by the plasma industry remaining as their objective. Roads that have “Work In Progress” signs (Figure 4) are still navigable, and PRT needs to be rapidly introduced whenever possible.

## Figures and Tables

**Table 1:** Transfusion transmitted malaria in Cameroon. From [9].

	Douala			Cameroon		
	Low	Medium	High	Low	Medium	High
**Population (estimation in 2010)**		1,907,479			19,406,100	
**Estimated N° of transfusions per year**	5150	8559	12,838	52,396	69,734	87,072
**Estimated N° of infections per year**	623	1061	1836	6550	20,049	39,182
**Probability for malaria transmission following blood transfusion-P (t)**	0.98	0.99	0.99	0.98	0.99	0.99

**Table 2:** Transfusion transmitted infections reported to the UK haemovigilance system 1996–2023. From [16].

	Bacteria	HAV	HBV	HCV	HEV	HIV	Malaria	Parvovirus B19	vCJD/ Prion	Total
**Total number of incidents (recipients)**	37 (40)	5	11 (14)	2	12 (15)	2 (4)	3	1	3 (4)	76 (88)

**Table 3:** Pathogen transmissions following blood component transfusion in the European Union [17].

Type of Transfusion Transmitted Infection	2022	2023
Bacteria	26	30
Viruses	5	9
Parasites	32	41

**Table 4:** Residual risk of transfusion of a single unit. From * [27] and ** [28].

Virus	Residual Risk per Million Units	
	1991 *	2025 **
HIV	6.7	0.23
HBV	25	0.87
HCV	5000 ***	0.16

*** Assumes no testing [as was the case when the reference was written].

**Table 5:** Summary of major pathogen-reduction technologies. Modified from [35].

PR Method (Brand Name)	Amotosalen + UVA (INTERCEPT)	Riboflavin + UV (MIRASOL)	Direct UVC 254 nm (THERAFLEX-UVC)	Methylene Blue/Visible Light (THERAFLEX-MB)
Type of technology/mechanism	Photochemical nucleic acid crosslinking using amotosalen, activated by UVA (320–400 nm), prevents replication; requires a compound adsorption device (CAD).	Photo-oxidation using riboflavin (vitamin B2); UV activates it; strand breaks and base modification by generated reactive oxygen species.	Chemical-free photolytic inactivation by direct UVC (254 nm); it induces pyrimidine dimers without additives.	Phenothiazine dye (methylene blue) absorbs visible light; singlet oxygen damages nucleic acids; filtration removes dye.
Applicable blood components	Platelets, plasma, cryoprecipitate and cryo-poor plasma licensed; RBC (amustaline + glutathione) in late-phase trials.	Plasma, platelets, and whole blood; RBC work ongoing.	Platelets developed, RBCs under investigation.	Plasma only; established in Europe.
Spectrum of pathogen inactivation	4–6 log for enveloped viruses/bacteria/parasites; lower for non-enveloped viruses.	3–6 log for viruses, 4–5 for bacteria, > 4 for parasites.	5–7 log for bacteria; broad viral coverage; less efficient if delayed treatment.	Strong for enveloped viruses; limited for non-enveloped and no bacterial action.
Impact on product quality	Slightly lower platelet CCIs (15–25% lower; minor reductions in factors; preserved hemostatic function. Autoantibodies to pathogen reduced red cells.	Comparable recovery; minimal biochemical changes; preserves RBC function in trials.	Faster metabolic deterioration; depends on bag and timing.	

## Data Availability

No new data were created or analyzed in this study.
